# Targeting senescent cells with NKG2D-CAR T cells

**DOI:** 10.1038/s41420-024-01976-7

**Published:** 2024-05-04

**Authors:** Yushuang Deng, Avadh Kumar, Kan Xie, Kristina Schaaf, Enzo Scifo, Sarah Morsy, Tao Li, Armin Ehninger, Daniele Bano, Dan Ehninger

**Affiliations:** 1 Translational Biogerontology Lab, German Center for Neurodegenerative Diseases (DZNE), Venusberg-Campus 1/99, 53127 Bonn, Germany; 2 Lonza Netherlands B.V., Geleen, Urmonderbaan 20-B, 6167 RD Geleen, Netherlands; 3 AvenCell Europe GmbH, Tatzberg 47, 01307 Dresden, Germany; 4 Department of Neurodegenerative Disease and Geriatric Psychiatry/Neurology, University of Bonn Medical Center, 53127 Bonn, Germany; 5 Aging and Neurodegeneration Lab, German Center for Neurodegenerative Diseases (DZNE), Venusberg-Campus 1/99, 53127 Bonn, Germany

**Keywords:** Senescence, Immunization

## Abstract

This study investigates the efficacy of NKG2D chimeric antigen receptor (CAR) engineered T cells in targeting and eliminating stress-induced senescent cells in vitro. Cellular senescence contributes to age-related tissue decline and is characterized by permanent cell cycle arrest and the senescence-associated secretory phenotype (SASP). Immunotherapy, particularly CAR-T cell therapy, emerges as a promising approach to selectively eliminate senescent cells. Our focus is on the NKG2D receptor, which binds to ligands (NKG2DLs) upregulated in senescent cells, offering a target for CAR-T cells. Using mouse embryonic fibroblasts (MEFs) and astrocytes (AST) as senescence models, we demonstrate the elevated expression of NKG2DLs in response to genotoxic and oxidative stress. NKG2D-CAR T cells displayed potent cytotoxicity against these senescent cells, with minimal effects on non-senescent cells, suggesting their potential as targeted senolytics. In conclusion, our research presents the first evidence of NKG2D-CAR T cells’ ability to target senescent brain cells, offering a novel approach to manage senescence-associated diseases. The findings pave the way for future investigations into the therapeutic applicability of NKG2D-targeting CAR-T cells in naturally aged organisms and models of aging-associated brain diseases in vivo.

## Introduction

Cellular senescence is marked by permanent cell cycle arrest and substantial macromolecular alterations in response to various stressors [1, 2]. Resistant to apoptosis, senescent cells persist in a viable and metabolically active state [3, 4]. This heightened metabolic activity of senescent cells is associated with the secretion of an array of proinflammatory cytokines, chemokines, and growth factors, collectively referred to as the senescence-associated secretory phenotype (SASP), which has emerged as an important contributor to the decline of tissue and organ function [5, 6]. The excessive accumulation of senescent cells has been reported in tissues of aged humans [7, 8], nonhuman primates [9, 10] and rodents [11, 12]. Noteworthy extensions of lifespan as well as improvements of aging-associated health outcomes [13], such as atherosclerosis [14], pulmonary dysfunction [15, 16], osteoarthritis [17], and cognitive impairments [18, 19], have been achieved by eliminating senescent cells using genetic or pharmaceutical approaches that induce senescent cells to undergo apoptosis (senoptosis) in vivo in several mouse models [13, 20, 21]. These findings indicate that senescent cells may represent a promising target for the development of new therapeutics and/or preventatives for age-related diseases. However, existing pharmacological approaches are not specific for senescent cells and are, hence, associated with side effects, which limits their translational potential [21].

To minimize the off-target effects of senotherapeutics, a promising alternative lies in immunotherapy-based targeting of senescence-specific surface antigens. For instance, a humanized antibody against dipeptidyl peptidase 4 (DPP4) [22] and an antibody-drug conjugate (ADC) directed at beta-2-microglobulin (B2MG) [23] have been demonstrated to effectively eliminate senescent cells expressing these antigens in vitro. Moreover, a vaccine targeting glycoprotein nonmetastatic melanoma protein B (GPNMB), another senescence-associated protein, was reported to improve age-related pathological changes in progeroid mice through the removal of senescent cells [24]. In addition to these antibody-based immune therapies, increasing evidence underscores the role of immune cells, including cytotoxic T cells, in the immunological surveillance of senescent cells in the context of cancer [25, 26]. Owing to their cytotoxic potency, effective migratory capacity, self-expansion, and memory abilities, T cells redirected with a chimeric antigen receptor (CAR) that targets specific antigens on cancer cells have received FDA approval for the treatment of various hematological malignancies, including lymphomas, certain leukemia, and multiple myeloma [27]. Recent advances have demonstrated that the adoptive transfer of CAR-T cells, specifically engineered to target the urokinase plasminogen activator receptor (uPAR)—a surface protein upregulated on some senescent cells—not only prolonged survival in a mouse model of lung adenocarcinoma undergoing oncotherapy-induced senescence, but also mitigated liver fibrosis triggered by chemical or dietary factors [28].

Although CAR-T cells exhibit enhanced precision in targeting senescent cells, the effectiveness of senolytic CAR-T therapies is largely dependent on accurately identifying senescence-associated surface antigens (senoantigens). Since senescent cells are heterogeneous and their properties are dynamic, identifying broadly expressed senoantigens remains challenging [29, 30]. Natural killer group 2D (NKG2D) is a type II transmembrane receptor expressed by NK cells and some subsets of T cells [31]. The expression of its ligands is increased in several tumor cell types and virus-infected cells in response to cellular stressors, while being generally absent in healthy cells [32]. Notably, consistent upregulation of NKG2D ligands (NKG2DLs) has been reported in various senescent cells in vitro [33–37] and senescent skin fibroblasts in elderly individuals in vivo [8]. Most recently, elimination of senescent cells by NKG2D-CAR T cells has been shown to improve several physiological changes and pathologies in both naturally aged and irradiated mice [37]. To take advantage of the stress-sensing ability of NKG2DLs, we developed NKG2D-CAR T cells and validated their targeting efficacy against NKG2DLs over-expressing B16F10 cell lines. Focusing on stress-associated senescence, we constructed genotoxic stress- and oxidative stress-induced senescence models using mouse forebrain astrocytes (AST) and mouse embryonic fibroblasts (MEFs). The broadly elevated expression of mouse NKG2DLs was established on the cell surface of these stress-related senescent cells. T cells engineered to express NKG2D-CAR exhibited selective and efficient cytotoxicity against NKG2DLs-expressing senescent AST and MEFs. Therefore, our data reinforce previous research and suggest that NKG2D-CAR T cells could serve as potent senolytics for aging and age-related diseases.

## Results

### Construction of NKG2D‑based CAR‑T cells

**Fig. 1:**
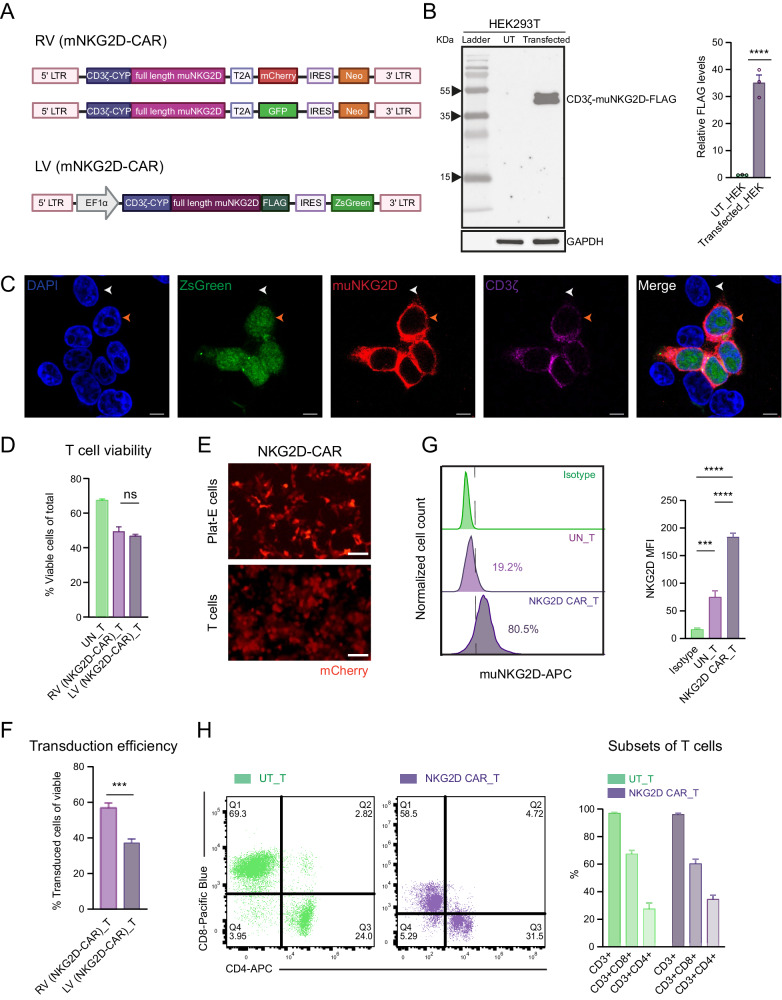
Construction of NKG2D‑based CAR‑T cells. **A** Schematic representation of retroviral (RV) and lentiviral (LV) NKG2D-CAR constructs encoding full-length mouse NKG2D receptor and cytoplasmic CD3ζ. **B** Western blot analysis of protein expression of FLAG-tagged NKG2D-CAR using anti-FLAG antibody in LV NKG2D-CAR construct-transfected and untransfected (UT) HEK293T cells. Data are presented as the mean ± SEM of three biological replicates; normalized to GAPDH protein level. ****P < 0.0001, determined by unpaired two-tailed Student’s t-test analysis. **C** Immunofluorescence co-staining of LV NKG2D-CAR construct-transfected HEK293T cells. Cells were co-stained with mouse NKG2D (red) and CD3ζ (purple) antibodies. Cell nuclei were stained with DAPI (blue). Orange and white arrows point to examples of NKG2D-CAR+ and NKG2D-CAR- cells, respectively. Scale bar = 10 μm. Cell viabilities (**D**) and transduction efficiencies (**F**) of RV and LV NKG2D-CAR-transduced T cells labelled with cell viability dye, analyzed by FACS, three to 4 days post-second round of transduction. The transduction efficiency was calculated as % mCherry/GFP/ZsGreen+ cells among viable cells. Data are presented as the mean ± SEM of six biological replicates. ns not significant, determined by one-way ANOVA followed by Tukey’s post-hoc tests. **E** Fluorescence microscopy images of packaging Plat-E cells (upper row) and T cells transduced with RV NKG2D-CAR 24 h post-second round of transduction (lower row). Scale bar = 200 μm. **G** Surface expression of NKG2D assessed by FACS. Histograms show the percentage of positive cells stained with APC-conjugated anti-mouse NKG2D antibody (purple) or isotype antibody (green). MFI of APC are presented as the mean ± SEM of six biological replicates. ***P < 0.001; ****P < 0.0001, determined by one-way ANOVA followed by Tukey’s post-hoc tests. **H** CD8 and CD4 subtypes of CD3 + UN-T cells and NKG2D-CAR T cells analyzed by FACS. NKG2D-CAR T cells were sorted by viable GFP+ or mCherry+ cells prior to analysis. Data are presented as the mean ± SEM of two biological replicates and determined by two-way ANOVA followed by Tukey’s post-hoc tests.

We generated NKG2D-CAR constructs by fusing full-length murine NKG2D to the CD3ζ cytoplasmic (CYP) domain (NKz-CAR) as previously described [38, 39], followed by GFP or mCherry expression cleaved via the Thosea asigna virus self-cleaving 2A peptide (T2A) skipping sequence based on a retroviral vector (RV) pFB. In the lentiviral vector (LV) pHAGE construct, NKz-CAR was accompanied by a FLAG tag and Zoanthus sp. green fluorescent protein (ZsGreen) expression, linked via an internal ribosome entry site (IRES) (Fig. 1A). The expression of NKG2D-CAR constructs was first validated in human embryonic kidney 293T (HEK293T) cells. Western blot analysis verified the expression of the FLAG-tagged fusion protein in LV NKG2D-CAR-transfected HEK293T cells (Fig. 1B). Immunofluorescence staining demonstrated the exclusive presence of NKG2D and CD3ζ in ZsGreen-positive HEK293T cells transfected with the LV NKG2D-CAR construct, further confirming the expression of the NKG2D-CAR construct (Fig. 1C). Activated pan T cells isolated from mouse spleen were then transduced with either RV or LV NKG2D-CAR. There was no significant difference in cell viability between RV and LV infected T cells, with a mean of 49.3% for RV (NKG2D-CAR) T cells and 46.8% for LV (NKG2D-CAR) T cells (Fig. 1D). Expression of the reporter protein mCherry was evident in packaging Plat-E cells (48 h-post transfection) and primary T cells (24 h-post the second round of transduction), as shown in Fig. 1E. The average transduction efficiency (% transduced viable cells) of RV (NKG2D-CAR) T cells (56.7%) was higher than that of LV (NKG2D-CAR) T cells (37.1%) (Fig. 1F). After three to 4 days of transduction, the surface expression of NKG2D receptor was evaluated by Fluorescence-Activated Cell Sorting (FACS). An increase in the percentage of NKG2D positive cells and a significant shift in mean-fluorescence intensity (MFI) beyond endogenous expression of NKG2D in native T cells were detected in T cells bearing the NKG2D-CAR (Fig. 1G).

To characterize T cell subpopulations, FACS was used to determine the proportions of CD4+ and CD8+ cells in both untransduced (UN)-T cells and NKG2D-CAR T cells. UN-T cells were harvested after activation and NKG2D-CAR T cells were obtained by sorting viable GFP+ or mCherry+ cells. Similar to UN-T cells, the majority (62.0% ± 2.5%) of NKG2D-CAR T cells were CD8+ cells, with no significant difference in the subsets of CD3+ T cells between UN-T cells and NKG2D-CAR T cells (Fig. 1H).

### Functional validation of NKG2D-CAR T cells

**Fig. 2:**
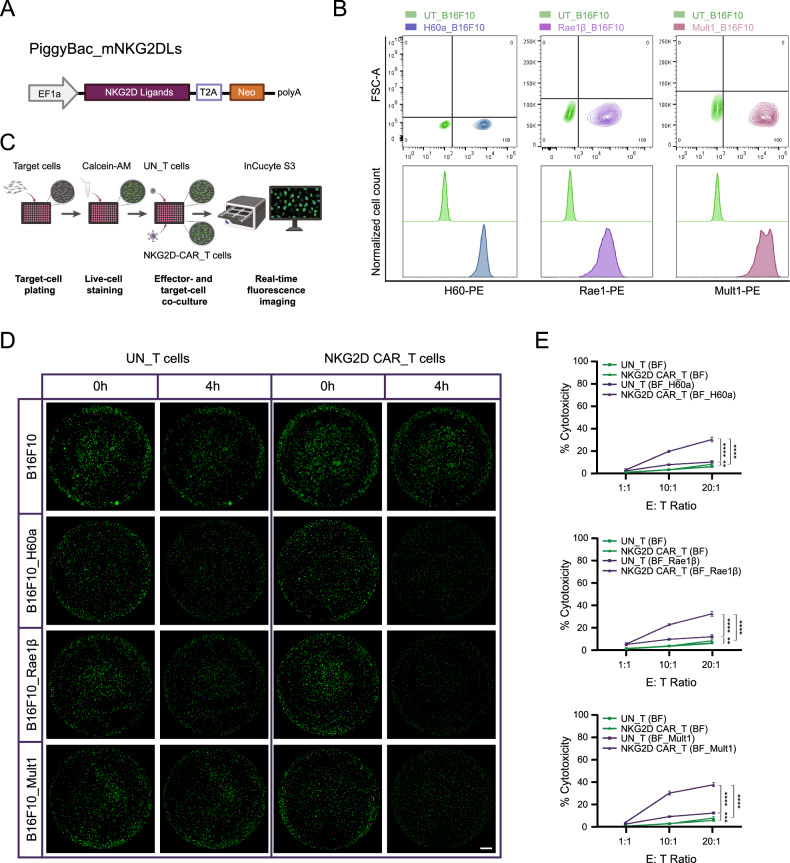
Functional validation of NKG2D-CAR T cells. **A** Schematic representation of PiggyBac construct encoding individual mouse NKG2DLs. **B** The surface expression of each mouse NKG2DL determined by FACS. Flow plots (upper panel) and histograms (lower panel) show the percentage of positive cells and MFI of PE-labeled mouse NKG2DL in UT-B16F10 cells (control) and NKG2DLs stably transfected B16F10 cells. The results are based on four independent technical experiments for each transfected cell colony. FSC-A, Forward-scatter area. **C** Schematic of Calcein-AM-based cytotoxicity assay. **D** Representative whole-well images of NKG2DL-transfected and UT-B16F10 target cells stained with Calcein-AM before (0 h) and after 4 h of co-culture with NKG2D-CAR T cells or UN-T cells at an E: T ratio of 20:1. Live cells labeled by Calcein-AM are shown in green. Scale bar = 800 μm. **E** Quantification of cytotoxicity of NKG2D-CAR T cells and UN-T cells against NKG2DLs over-expressing B16F10 cells and B16F10 control target cells at E: T ratios of 1:1, 10:1, and 20:1 after 4 h of co-culture. Data are presented as the mean ± SD of three biological replicates. **P < 0.01; ***P < 0.001; ****P < 0.0001, determined by two-way ANOVA followed by Tukey’s post-hoc tests. BF, B16F10.

To assess the efficacy with which NKG2D-CAR T cells can target NKG2DL-expressing cells, we first established, using the PiggyBac transposon system, B16F10 melanoma cells individually overexpressing one of three classes of mouse NKG2DLs: histocompatibility antigen 60a (H60a), retinoic acid early transcript 1β (Rae1β) or murine UL16-binding-protein-like transcript 1 (Mult1) (Fig. 2A). Following neomycin selection, individual colonies were picked and expanded into stably transfected cell lines. The surface expression of each inserted NKG2DL was confirmed by FACS. Unmodified B16F10 cells, known to lack NKG2DLs [40, 41], served as control cells. For each NKG2DL, a dramatic right shift in MFI was observed in transfected B16F10 cell lines compared to the negative control (Fig. 2B).

Next, we performed cytotoxicity assays using NKG2D-CAR T cells or UN-T cells against NKG2DL-expressing B16F10 cells at different effector-to-target (E: T) ratios. Target cell viability was dynamically evaluated using green fluorescent Calcein-AM labeling of live cells in conjunction with automated longitudinal microscopy (Fig. 2C). Untransfected (UN) B16F10 cells served as negative controls. After 4 h of co-culture, the % cytotoxicity for each sample was calculated using the formula (1-live fluorescent cell count at 4 h/live fluorescent cell count at 0 h) × 100. The results demonstrated that NKG2D-CAR T cells exhibited dose-dependent cytotoxicity against all three classes of NKG2DL-overexpressing cells, with maximum cell killing efficacies of 29.9%, 32.9%, and 37.2%, respectively, at an E: T ratio of 20:1. Conversely, NKG2D-CAR T cells displayed minimal cytotoxicity (up to 8.0%) against NKG2DL-negative control cells (Fig. 2D, E). Even at the highest E: T ratio, UN-T cells with endogenous NKG2D receptor expression exhibited limited cytotoxic activity towards NKG2DL-expressing target cells (up to 12.5% lysis) compared to baseline (5.9% lysis of UN-T cells against control cells) (Fig. 2D, E).

### NKG2D ligands are expressed in senescent cells induced by DNA damage and oxidative stress

**Fig. 3:**
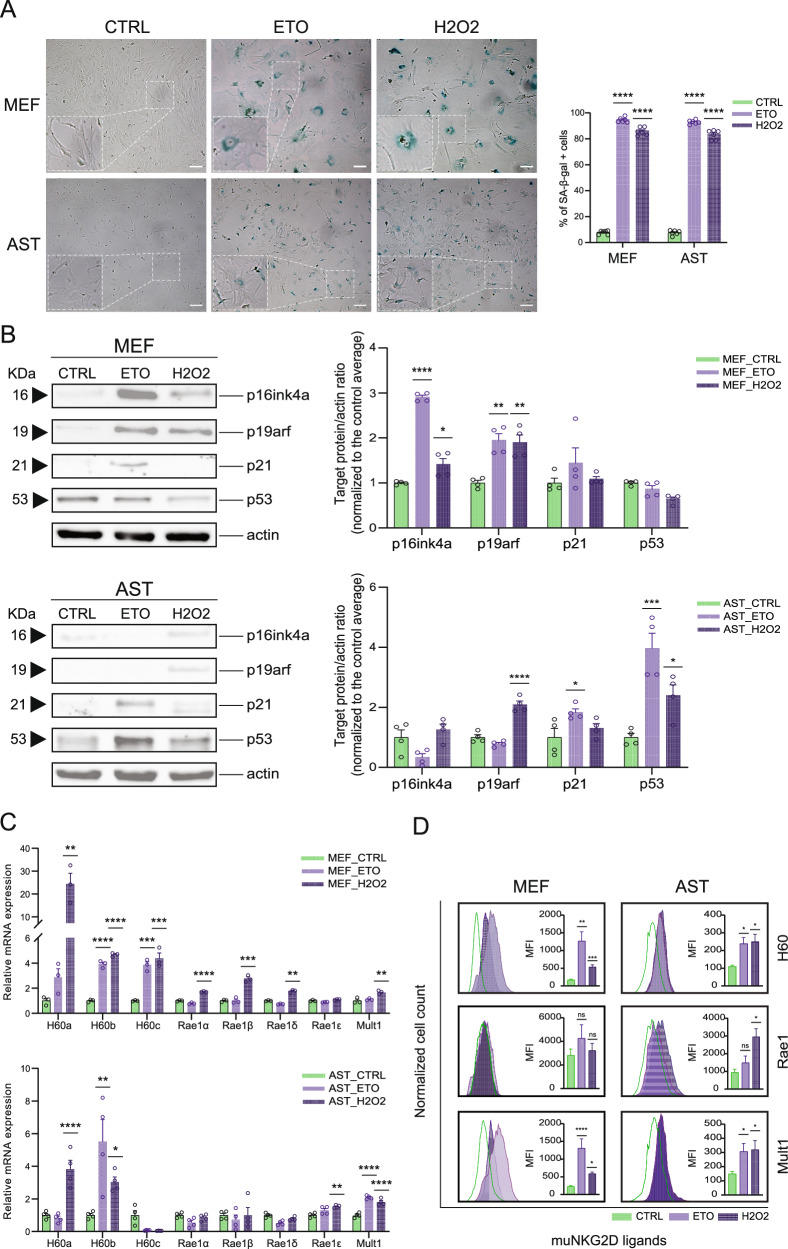
NKG2D ligands are expressed in senescent cells induced by DNA damage and oxidative stress. **A** Representative images of SA-β-gal staining of MEFs (upper panel) and AST (lower panel) treated with or without ETO or H2O2 visualized under bright-field microscopy. Blue cells indicate SA-β-gal positive cells. Scale bar = 100 μm. Quantification of % SA-β-gal positive cells was performed in randomly selected view fields. Data are presented as the mean ± SEM of six biological replicates. **B** Western blot analysis (left panel) and quantification (right panel) of protein expression of senescence markers in MEFs and AST with or without ETO or H2O2 treatment. The band intensity of a given target protein was normalized to the corresponding actin signal for each sample. Data were normalized to the average of the corresponding control group and are presented as the mean ± SEM, based on n = 4 biological replicates. Statistical significance was assessed using one-way ANOVA followed by Tukey’s post-hoc tests, where appropriate, to compare each senescence marker within a specific cell type across various conditions (control and treatments). **C** qPCR analysis of mRNA expression of mouse NKG2DLs in senescent MEFs and AST induced by ETO or H2O2 treatment. Data are presented as the mean ± SEM of three or four biological replicates; normalized to β-actin mRNA level. **D** Cell surface expression of each mouse NKG2DL in senescent MEFs and AST (filled purple) compared to the corresponding proliferative control cells (green lines) determined by FACS. MFI of PE-labeled mouse NKG2DLs are presented as mean ± SEM of three biological replicates. *P < 0.05; **P < 0.01; ***P < 0.001; ****P < 0.0001, determined by one-way ANOVA followed by Tukey’s post-hoc tests (**A**–**D**).

NKG2DLs are typically absent on healthy cells, and their elevated expression is linked to various cellular stressors [32]. To evaluate whether NKG2DLs expression is induced during stress-associated senescence, MEFs and mouse primary forebrain AST were treated with the nuclear DNA damage reagent etoposide (ETO) and the reactive oxygen species (ROS) reagent hydrogen peroxide (H2O2) to induce senescence. Senescence was verified by measuring several common senescence biomarkers. Senescent MEFs and AST induced by either treatment displayed flattened and enlarged morphologic changes and increased positive staining for senescence-associated beta-galactosidase (SA-β-gal) compared to proliferative control cells (Fig. 3A). Elevated protein levels of p16ink4a and p19arf were detected in the senescent MEFs, and increased protein expression of p19arf, p21, and p53 was determined in senescent AST (Fig. 3B).

The mRNA and surface protein expression of mouse NKG2DLs in these senescent cells were then measured by qPCR (Fig. 3C) and FACS (Fig. 3D), respectively. Notably, a significant increase in H60 and Mult1 protein levels was detected in all senescent cells, regardless of cell type and inducer (Fig. 3D). Rae1 was enhanced in H2O2-treated MEFs at the mRNA level (Fig. 3C) and in H2O2-treated AST at both mRNA and protein levels (Fig. 3C, D). The observations outlined above suggest that NKG2DLs may serve as potential targets for CAR-T cell-mediated elimination of stress-associated senescent cells.

### NKG2D-CAR T cells exhibit robust cytotoxicity against stress-induced senescent MEFs and AST in vitro

**Fig. 4:**
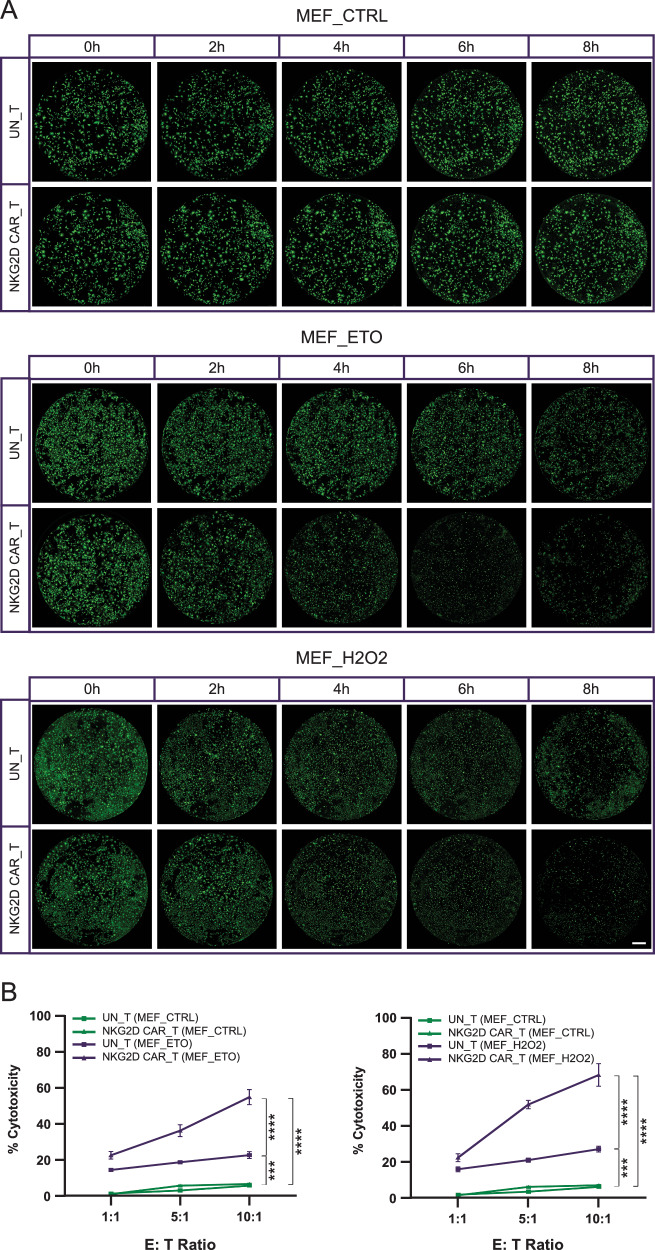
Cytotoxicity of NKG2D-CAR T cells against stress-induced senescent MEFs. **A** Representative whole-well images of senescent MEFs induced by ETO or H2O2 treatment and untreated proliferative MEFs, stained with Calcein-AM, captured every 2 h during the 8 h of co-culture with NKG2D-CAR T cells or UN-T cells at an E: T ratio of 10:1. Live cells were labeled by Calcein-AM in green. Scale bar = 800 μm. **B** Quantification of cytotoxicity of NKG2D-CAR T cells and UN-T cells against senescent MEFs and proliferative MEFs at E: T ratios of 1:1, 5:1, and 10:1 after 8 h of co-culture. Data are presented as the mean ± SD of four biological replicates. ***P < 0.001; ****P < 0.0001, determined by two-way ANOVA followed by Tukey’s post-hoc tests.

**Fig. 5:**
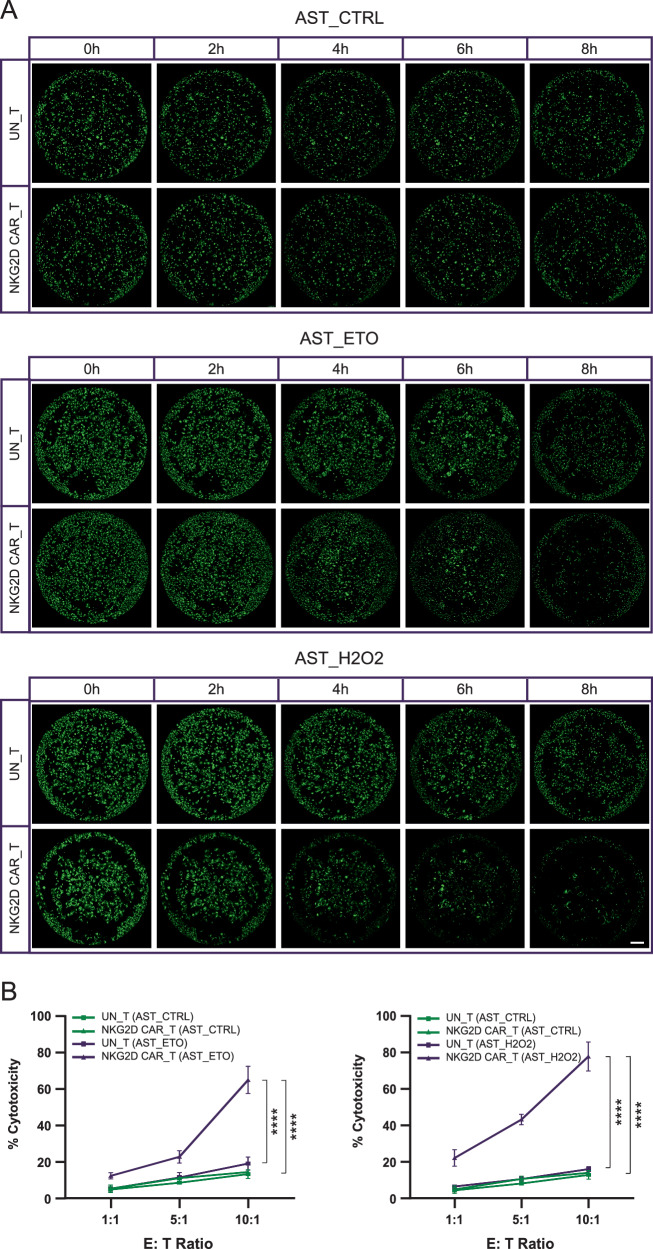
Cytotoxicity of NKG2D-CAR T cells against stress-induced senescent mouse AST. **A** Representative whole-well images of senescent AST induced by ETO or H2O2 treatment and untreated proliferative AST, stained with Calcein-AM, captured every 2 h during the 8 h of co-culture with NKG2D-CAR T cells or UN-T cells at an E: T ratio of 10:1. Scale bar = 800 μm. **B** Quantification of cytotoxicity of NKG2D-CAR T cells and UN-T cells against senescent AST and proliferative AST at E: T ratios of 1:1, 5:1, and 10:1 after 8 h of co-culture. Data are presented as the mean ± SD of four biological replicates. ****P < 0.0001, determined by two-way ANOVA followed by Tukey’s post-hoc tests.

To assess the killing capacity of NKG2D-CAR T cells against senescent cells expressing endogenous NKG2DLs in vitro, we co-cultured NKG2D-CAR T cells or UN-T cells with proliferative or senescent MEFs or AST for 8 h at different E: T ratios of 1:1, 5:1, and 10:1. A Calcein-AM based cytotoxicity assay was employed to determine the lytic activity of effector T cells (Figs. 4A, 5A). Substantial increases in cytotoxicity of NKG2D-CAR T cells compared to UN-T cells against both ETO-triggered and H2O2-induced senescent MEFs were observed in a dose-dependent manner, with maximum specific killing efficacies of 53.9% vs. 21.9% and 67.3% vs. 26.0%, respectively, at an E: T ratio of 10:1 (Fig. 4). Conversely, NKG2D-CAR T cells induced minimal lysis of proliferative cells, with a maximum of 6.3% across all E: T ratios (Fig. 4), indicating the limited off-target activity of NKG2D-CAR T cells. A similar cytolytic profile was observed for NKG2D-CAR T cells against senescent AST, with the maximum specific cytolysis reaching 78.7% at an E: T ratio of 10:1 when targeting the senescent AST induced by oxidative stress (Fig. 5). UN-T cells displayed limited cytotoxicity, up to 18.4%, towards senescent AST regardless of the inducer, which is comparable to the killing effect of NKG2D-CAR T cell against proliferative AST (less than 15%) (Fig. 5). In summary, our cytotoxicity data indicate that T cells bearing NKG2D-CAR selectively eliminate NKG2DL-expressing senescent cells, induced either via DNA damage or oxidative stress.

## Discussion

Given that the accumulation of senescent cells is thought to contribute to a wide range of age-related changes and pathologies, there is growing interest in the development of therapeutics that are based on the removal senescent cells from tissues [3, 6, 21, 42, 43]. Notably, immunotherapeutic strategies, widely employed for targeting specific cancer antigens, have also been adapted to tackle senescent cells [44]. T cells engineered with synthetic CARs that enhance T cell specificity and cytolytic capacity have shown significant efficacy in patients with hematological malignancies [27]. However, this efficacy appears suboptimal in the context of solid tumors [45]. Unlike tumor cells, senescent cells do not create an immunosuppressive microenvironment that interferes with T cell activity [46, 47]. Moreover, the senescent cell-specific secretome, known as SASP, may aid in the trafficking and infiltration of adoptive CAR-T cells [48], potentially amplifying the effectiveness of this cell-based immunotherapy in the context of addressing senescence-associated diseases. Indeed, existing evidence suggests that CAR-T cells targeting uPAR-expressing senescent cells in lung adenocarcinoma and liver fibrosis have been effective in mouse models [28], indicating that senoantigen-targeting CAR-T cells may provide viable strategies for eliminating senescent cells from tissues.

The efficacy and safety of CAR-T cells vary based on CAR design [49]. Although the NKz-CAR design does not incorporate commonly used costimulatory genes, like CD28 and CD137, which are known to enhance T cell effector function and persistence [49], the NKG2D receptor in this configuration naturally associates with the costimulatory molecules DNAX-activating protein 10 (DAP10) in humans and/or DAP12 in mice. This association transduces a costimulatory signal via the phosphatidylinositol 3-kinase (PI3K) pathway in T cells, suggesting that NKz-CAR activates T cells using both CD3ζ and a costimulatory signal through DAP10/DAP12, thereby functioning as the second generation of CARs [50, 51]. In this study, NKz-CAR T cells effectively target NKG2DL-expressing B16F10 cells, which is consistent with the elimination of NKG2DL–positive tumor cells by similarly modified human and mouse T cells in previous studies [38, 39, 52]. This validates the binding affinity of our NKz-CAR. The low expression levels of human NKG2DLs (MHC class I chain-related proteins A (MICA) and MICB) on normal intestinal epithelial cells [53] and the detection of mouse ligand Mult1 in several normal tissues [54] could result in off-target effects under NKG2D-CAR T cell therapy. Therefore, it is crucial to achieve optimal binding affinity in NKG2D-CARs, allowing the CARs to effectively engage their targets and execute T cell effector functions, while simultaneously minimizing on-target but off-tumor effects, especially when targeting ligands that are expressed at low levels on healthy cells. Of note, severe toxicity was noted in BALB/c and C57BL/6 mice with DAP10 or CD28 fused-NKz-CAR T cells, but not with NKz-CAR T cells alone [55]. This suggests that the short-term persistence of NKz-CAR T cells may be advantageous in minimizing off-target effects. In addition, our NKz-CARs, based on the natural extracellular recognition domain of the NKG2D receptor, would be less likely to be immunogenic in vivo compared to CARs expressing a single-chain fragment variable. Encouraging evidence on the favorable safety of NKz-CAR T cells has been demonstrated in clinical trials in cancer therapy [56]. Recent studies involving T cells engineered with CARs, which incorporate the NKG2D extracellular domain, the CD8 transmembrane domain, and an additional CD137 costimulatory domain instead of CD28, have demonstrated an absence of side effects in aged nonhuman primates [37]. The optimal efficacy and safety profile of senolytic NKz-CAR T cells should be further addressed in future research.

Astrocytes, the most predominant glial cell type in the brain, play a crucial role in central nervous system physiology, providing structural support and engaging in various metabolic activities [57]. The accumulation of senescent astrocytes has been documented in both aged human brains [58] and those of Alzheimer’s disease (AD) patients [59]. Genetically removing p16-expressing senescent glial cells has been demonstrated to alleviate cognitive decline, reduce tau hyperphosphorylation, and prevent gliosis in a tauopathy mouse model [18]. Consistent with prior work [60, 61], our study showed that mouse forebrain astrocytes undergo senescence in vitro in response to both genotoxic and oxidative stress. Interestingly, astrocytes demonstrated a higher sensitivity to both DNA damage and oxidative stress compared to MEFs, as indicated by the lower doses and shorter durations required to induce senescence. This establishes stress-triggered senescence in astrocytes as a promising model for exploring cellular senescence within the context of the brain.

While p16 and p21 are widely recognized as cellular markers for senescent cells, due to their roles in governing cell cycle arrest, previous cell culture studies have reported their distinct functions in inhibiting cell proliferation [2, 62]. The p53/p21 pathway is believed to be activated early in the senescence program, primarily sensing dysfunctional telomeres as damaged DNA. In contrast, the p16/RB pathway is thought to play a more substantial role in maintaining the senescent state independent of telomere status [62]. Recent in vivo single-cell transcriptomic analysis revealed that cells highly expressing p21 and those highly expressing p16 represent two distinct populations in various aged tissues [63]. In our study, DNA damage and oxidative stress in mouse astrocytes led to elevated p21/p53 expression without activating p16. This observation, combined with the extensive activation of cell cycle regulators (including p16, p53, and p21) in human astrocytes following H2O2 treatment [60], and the lack of these characteristics in rat astrocytes under DNA damage stress [64], suggests that the features of cell cycle regulators in astrocyte senescence are not conserved across experimental conditions.

Compared to the divergent expression of these classical senescence-associated cell cycle regulators, increased expression of NKG2DLs may serve as a general hallmark of senescence. This hallmark has been identified in a variety of senescence models originating from diverse cell types and inducers [33–37]. These models include replicative senescent human fibroblasts [33, 34] and human umbilical vein endothelial cells [35], oncogene‐induced senescent human fibroblasts [33], chemotherapy-induced senescent human and mouse fibroblasts [33, 34, 37], ultraviolet irradiation-triggered senescent mouse fibroblasts [37], and chemotherapy-induced senescent human hepatic stellate cells [33, 34]. Moreover, in elderly human individuals, senescent dermal fibroblasts in vivo exhibit high expression levels of NKG2DLs. In our study, a universal increase in the protein levels of mouse NKG2DLs, including H60 and Mult1, was observed during senescence, irrespective of cell type or senescence inducing-stimulus. This widespread elevation underscores the potential significance of NKG2DLs as a shared feature in senescent cells, contributing to their immunogenicity across diverse contexts.

NKG2D-CAR T cells targeting NKG2DLs have shown anticancer effects against various tumor types, such as T-cell lymphoma [39], glioblastoma [52], osteosarcoma [65], and colorectal cancer [66]. To assess the effectiveness of NKz-CAR T cells against in vitro models of NKG2DLs-expressing senescent cells, we conducted a Calcein-AM-based cytotoxicity assay using the IncuCyte platform. This approach enabled real-time kinetic monitoring of cytotoxicity throughout the duration of co-culture. Our methodology offers advantages over previously used endpoint assays, ensuring a more accurate and dynamic assessment of potency [67]. Our findings revealed that NKz-CAR T cells selectively eliminated senescent MEFs and mouse AST induced by either DNA damage or oxidative stress while displaying limited lytic ability towards proliferative cells lacking NKG2DLs. A recent study [37] observed that CD137-fused NKG2D-CAR T cells targeting ETO-induced senescent MEFs exhibited comparable cytolytic activity, but at a significantly lower E: T ratio of up to 2:1. This outcome could be attributed to the enhanced efficacy of effector cells resulting from additional co-stimulation.

In addition to the efficacy of CAR-T cells, antigen density is another determinant influencing the cytotoxic activity of these cells [49]. However, our study did not identify a correlation between NKG2DLs expression levels and NKz-CAR T cell mediated cytotoxicity, as evidenced by the similar cytotoxicity of NKz-CAR T cells towards senescent MEFs and AST despite their differing patterns of increased NKG2DLs expression. This finding is consistent with similar results demonstrated in various tumor studies [38, 65, 68], suggesting that different NKG2DLs expression profiles in different cell contexts do not necessarily dictate the responsiveness of NKG2D-CAR T cells. The variation in binding affinities of NKG2D-CAR T cells to its different ligands, along with the different abilities of NKG2DLs to elicit downstream activating signals, may contribute to this lack of correlation [69]. Moreover, although human NKG2DLs lack homology with mouse ligands based on the sequence comparison, the stress-sensing ability of NKG2DLs is conserved across both species [50]. Validation of the effectiveness of human NKG2D-based CAR-T cells targeting senescence in human cells is crucial for advancing NKG2D-CAR T cell-based clinical senotherapy.

The subsets of CD8+ and CD4+ T cells within the CAR-T cells could also affect the cytolytic activities [70]. There is increasing evidence for the comparable effectiveness of CD4+ CAR-T cells in killing target tumor cells compared to their CD8+ counterparts [71–73]. Although a longer conjunction period and delayed kinetics is required for CD4+ CAR-T cells, they are less prone to exhaustion or activation-induced cell death [74]. Furthermore, T cells expressing CD19-CAR, originating from a defined 1:1 ratio of CD8:CD4 CAR-T cells, have shown superior antitumor activities in vivo [75, 76]. In our study, the subsets of NKz-CAR T cells comprised an average of 62% CD8+ T cells and a substantial proportion of CD4+ T cells (average 33.3%). Since most clinical trials and preclinical experiments use randomly composed CD8+ and CD4+ CAR-T cell subsets [70], further studies are warranted to determine the optimal composition of CD8+ and CD4+ NKG2D-based CAR-T cells for targeting senescence, both in vitro and in aged individuals in vivo.

**Fig. 6:**
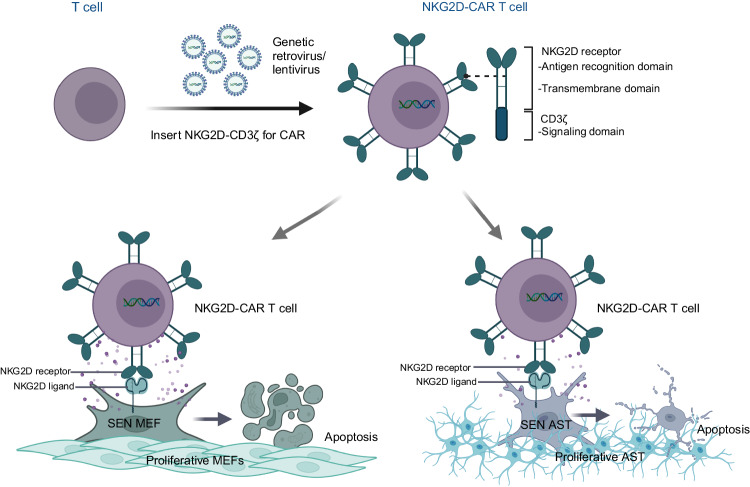
Selective elimination of NKG2DLs-expressing senescent MEFs and mouse AST by NKG2D-CAR T cells. NKG2D-CAR consists of an antigen recognition domain-NKG2D receptor fused to a CD3ζ cytoplasmic signaling domain. T cells are engineered with NKG2D-CAR-encoding retroviral or lentiviral vectors. Genotoxic insults and oxidative stress induce surface expression of NKG2DLs on MEFs and AST. Activated NKG2D-CAR T cells specifically recognize NKG2DLs expressed on the surface of senescent MEFs and AST, subsequently exerting cytotoxic effects on the target cells. SEN senescence.

In summary, our study provides preliminary preclinical evidence of the efficacy of NKG2D-CAR T cells in eliminating stress-induced senescent cells in vitro (Fig. 6). Given that senescence is linked to a range of pathological changes in aging and aging-related diseases [43], our findings suggest a broad clinical therapeutic potential of senolytic CAR-T cells. Here we present the first proof of cytolytic activities of NKG2D-CAR T cells in senescent astrocytes, indicating targetability of senescent brain cells using this approach. Additional research is required to more comprehensively understand the therapeutic potential of NKG2D-targeting CAR-T cells in naturally aged organisms and in vivo models of aging-associated diseases.

## Materials and methods

### Antibodies

The following antibodies were used for Western blot: Rabbit anti‐FLAG (1:1 000, Cell Signaling Technology, Danvers, MA, USA, #14793), rabbit anti-CDKN2A/p16INK4a (1:1000, Abcam, Cambridge, UK, #ab211542), rat anti-CDKN2A/p19ARF (1:1000, Abcam, #ab26696), rabbit anti-p53 (D2H9O) (1:1 000, Cell Signaling Technology, #32532 S), rabbit anti-p21 Waf1/Cip1 (1:1000, Cell Signaling Technology, #64016S), mouse anti‐actin (1:5000, MP Biomedicals, Solon, OH, USA, #SKU 0869100), mouse anti‐glyceraldehyde-3-phosphate dehydrogenase (GAPDH) (1:3000, Abcam, #ab8245). Horseradish peroxidase-conjugated secondary antibodies were purchased from Promega, Madison, WI, USA (1:3000, goat anti-rabbit), Agilent Technologies, Santa Clara, CA, USA (1:10,000, goat anti-mouse), and Cell Signaling Technology (1:2000, goat anti-rat). The following antibodies were used for immunofluorescence: rat anti-glial fibrillary acidic protein (GFAP) (1:200, Thermo Fisher Scientific, Waltham, MA, USA, #13-0300), rat anti-mouse NKG2D/CD314 (1:500, R&D Systems, Minneapolis, MN, USA, #MAB1547-100), rabbit anti-CD3 zeta (1:250, BIOZOL, Eching, Germany, #ABN-H00000919-D01P). Alexa Fluor-conjugated secondary antibodies, including goat anti-rat IgG Alexa Fluor 488 (1:500, #A-11006), donkey anti-rat IgG Alexa Fluor 594 (1:500, #A-21209), goat anti-rabbit IgG Alexa Fluor 647 (1:500, #A-21245) were obtained from Thermo Fisher Scientific. The following antibodies were used for Flow cytometry: Allophycocyanin (APC)-conjugated anti-mNKG2D antibody (1:100, eBioscience, San Diego, CA, USA, #17-5882-82), APC-conjugated-rat IgG1 isotype control (1:100, eBioscience, #17-4301-82), FITC-conjugated anti-mCD3 (1:50, Miltenyi Biotec, Bergisch Gladbach, Germany, #130-119-798), APC-conjugated anti-mCD4 (1:50, Miltenyi Biotec, #130-116-487), VioBlue-conjugated anti-mCD8a (1:50, Miltenyi Biotec, #130-102-431), Phycoerythrin (PE)-conjugated anti-mH60 antibody (1:50, Miltenyi Biotec, #130-108-820), PE-conjugated anti-mRae-1 Pan antibody (1:50, Miltenyi Biotec, #130-111-283), PE-conjugated anti-mMult1 antibody (1:100, R&D systems, #FAB2588P).

### Mice

C57BL/6J wild-type (strain #000664) mice were purchased from The Jackson Laboratory. All animals were group housed under specific pathogen-free conditions. Mice were kept on a 12:12 h light-dark cycle at a constant temperature of 22 °C and 55% humidity. Food and water were supplied at libitum. In accordance with the German Animal Welfare Act, the present study was approved by the “Landesamt für Natur, Umwelt und Verbraucherschutz Nordrhein-Westfalen” (Recklinghausen, Germany). Local and federal animal welfare regulations were followed. Adult animals were sacrificed by CO2 suffocation.

### Cell culture

HEK293T cells and B16F10 murine melanoma cells (LGC Standards, Middlesex, UK, #ATCC-CRL-6475) were cultured in Dulbecco’s modified Eagle’s medium (DMEM, Thermo Fisher Scientific) supplemented with 10% fetal bovine serum (FBS, Thermo Fisher Scientific) and 1% penicillin-streptomycin (Thermo Fisher Scientific). Primary MEFs were isolated from E13.5 wild-type C57BL/6J mouse embryos as previously described [77], and cultured in DMEM supplemented with 10% FBS and 1% penicillin-streptomycin. Primary astrocytes from mouse forebrains were prepared as previously described [78]. Early passages (P1–P3) of isolated astrocytes were then validated by staining of the astrocyte marker GFAP (Supplementary Fig. 1). Astrocytes were cultured in DMEM supplemented with 10% FBS and 1% penicillin-streptomycin. All cells were maintained at 37 °C in a humidified 5% CO2 atmosphere.

### Viral NKG2D-CAR vectors and PiggyBac NKG2DLs vector construction

Mouse NKG2D-CAR was engineered by fusing the murine CD3ζ CYP region coding sequence (CD3ζ-CYP) to the full-length coding sequence of murine NKG2D receptor (NKz-CAR), as previously described [38, 39]. The NKz-CAR cDNA was synthesized by Genscript (Piscataway, NJ, USA, www.genscript.com) and procured as a cloned construct in the pUC57 cloning vector. NKz-CAR was amplified by polymerase chain reaction (PCR). Primers used are listed in Supplementary Table 1. The retroviral NKz-CAR was generated based on pFB-internal ribosomal entry site (IRES)-Neo (neomycin resistance) retroviral vector backbone (Addgene, Watertown, MA, USA, #69767), which was modified by inserting the mCherry/GFP sequence linked by a T2A skipping sequence to induce co-expression of a reporter protein. NKz-CAR was then subcloned into pFB-T2A-mCherry/GFP-IRES-Neo vector. Similarly, the lentiviral NKz-CAR was constructed based on the pHage-EF1α (elongation factor 1-alpha)-MICA-IRES-ZsGreen lentiviral vector backbone (Addgene, #114007), in which MICA was replaced by NKz-CAR with restriction enzyme-based cloning.

To generate PiggyBac constructs encoding individual mouse NKG2DLs, the PiggyBac transposon vector backbone containing 5’ inverted terminal repeat (5’ ITR) and 3’ ITR sequence was custom-synthesized by VectorBuilder (vectorbuilder.com) and modified by inserting the EF1α promoter sequence and Neo sequence followed by terminator sequence SV40 PolyA (simian virus 40 PolyA), linking with T2A. The coding sequences of murine NKG2DLs were PCR-amplified from commercially synthesized constructs pCMV6-Ampicillin-IRES-GFP containing H60a, Rae1β or Mult1 (Genscript). Primers used are listed in Supplementary Table 1. The PCR products were cloned into the modified PiggyBac transposon expression vector PB-EF1α-T2A-Neo-PolyA, yielding PB-EF1α-NKG2DL-T2A-Neo-PolyA constructs encoding individual mouse NKG2DLs driven by the EF1α promoter. All plasmids were digested using restriction enzymes (New England BioLabs, Ipswich, MA, USA) at 37 °C for 1 h and ligated using the T4 DNA Ligase (New England BioLabs, #M0202L). The plasmids were transformed into E.coli DH5α cells (Thermo Fisher Scientific, #18265017) for propagation, and plasmid DNA was extracted using the GeneJET Plasmid Miniprep Kit (Thermo Fisher Scientific, #10242490) following the manufacturer’s protocol and verified by sequencing (Eurofins Genomics, eurofinsgenomics.eu).

### Cell transfection and stable cell lines establishment

HEK293T cells were transiently transfected with lentiviral NKz-CAR plasmid using Lipofectamine 3000 (Thermo Fisher Scientific, #L3000001) according to the manufacturer’s instructions and analyzed for expression of both NKG2D-CAR and the FLAG-tag 48 h post-transfection. To establish stable B16F10 cell lines expressing individual mouse NKG2DLs, B16F10 cells were co-transfected with the PiggyBac transposon plasmid PB-EF1α-NKG2DL-T2A-Neo-PolyA and PiggyBac transposase expression vector (System Biosciences, Palo Alto, CA, USA, #PB210PA-1) using Lipofectamine 3000. The cells were then selected with 2 mg/ml of G418 (Omnilab, Bremen, Germany, #1198774) and single clones were picked. After expansion, the individual clones were analyzed for the expression of the respective mouse NKG2DL.

### Mouse T cell isolation and transduction

Spleens from 8–12-week-old wild-type C57BL/6J mice were harvested. Following tissue dissection and red blood lysis (1X RBC Lysis Buffer, eBioscience, #00-4333-57), primary mouse Pan T cells were purified using the CD90.2 microbeads (Miltenyi Biotec, #130-121-278) by magnetic-activated cell sorting (MACS) according to the manufacturer’s protocol. Purified T cells were cultured in RPMI-1640 (Thermo Fisher Scientific) supplemented with 10% FBS, 1% penicillin-streptomycin, 55 µM β-mercaptoethanol (Thermo Fisher Scientific), 1X ITS (Merck, Darmstadt, Germany, #13146), 80 IU/ mL of recombinant human IL-2 (rhIL-2) (Bio-Techne, Minneapolis, MN, USA, #202-IL-010). Mouse T cells were activated with 5 µg/ml concanavalin A (Merck, #C2010). Prior to plating T cells, a 12-well plate was coated with 15 μg/ml Retronectin (TaKaRa, Shiga, Japan, #T100A) at 4 °C overnight. The Retronectin solution was aspirated the following day, then the plate was coated with 2% bovine serum albumin (BSA, Carl Roth, Karlsruhe, Germany) for 30 min at room temperature. Activated mouse T cells (not later than 24 h) were resuspended at 1.5 × 10^6^ cells/ml in RPMI-1640 medium containing 10% FBS and 80 IU/ mL of rhIL-2. The supernatants of “Platinum-E” (Hölzel Diagnostika, Cologne, Germany, #RV-101) packaging cells transfected with retroviral NKz-CAR plasmid and helper plasmid pCL-Eco (Addgene, #12371) using Lipofectamine 3000 were harvested 48 h post-transfection for retroviral infection. Lentiviral particles were collected from the supernatants of “Platinum-A” (Hölzel Diagnostika, #RV-102) packaging cells transfected with lentiviral NKz-CAR plasmid and helper plasmids pCMV-dR8.2 dvpr (Addgene, #8455) and pCMV-VSV-G (Addgene, #8454), and concentrated by polyethylene glycol (BIOZOL, #LV825A-1) according to the manufacturer’s protocol. The virus-containing supernatants were filtered through 0.45 μm filters. For retrovirus infection, 4 μg/ml polybrene (Merck, #TR-1003-G) was added into retroviral supernatants. Then, a 1:1 vol/vol ratio of activated mouse T cells and retroviral supernatant-polybrene were plated into a 12-well plate coated with Retronectin, followed by centrifugation at 2000 × *g*, 30 °C for 1 h. The second round of transduction was conducted with the retroviral supernatants collected 72 h-post transfection by centrifugation at 2000 × *g*, 30 °C for 1 h the following day after the first round of transduction. For lentivirus infection, the concentrated virus was added to the activated mouse T cells at a multiplicity of infection of 50. After transduction, cells were expanded in the fresh medium supplemented with rhIL-2. Culture medium was changed every other day, and the cells were counted daily and maintained at 1–2 × 10^6^ cells/ml. The transduced T cells were used for expression and functional analysis three to 4 days post-transduction.

### Western blot

Cell pellets were lysed in Tris-Buffered Saline (TBS, pH7.6) (Merck) supplemented with 2% SDS (Carl Roth), 1x Protease Inhibitor Cocktail (Roche, Mannheim, Germany) and 1x PhosSTOP Phosphatase Inhibitor Cocktail (Roche). 30–50 mg protein samples were separated on 10–15% SDS-PAGE gels (according to their molecular weight) and transferred onto nitrocellulose membranes (GE Healthcare, Chicago, IL, USA). Subsequently, the membranes were blocked with 10% skim milk in Phosphate-Buffered Saline (PBS) for 1 h at room temperature and incubated with primary antibodies overnight at 4 °C. After washing in PBS containing 0.1% Tween20 (Carl Roth), the membranes were incubated with Horseradish peroxidase-conjugated secondary antibodies for 1.5 h at room temperature. Immunoblots were imaged using a ChemiDoc Imager (Bio-Rad, Hercules, CA, USA), and band intensities were quantified with ImageJ software.

### Immunofluorescence

Cells were fixed in 4% paraformaldehyde (Santa Cruz Biotechnology, Dallas, TX, USA) for 10 min, washed with ice-cold PBS for three times, and permeabilized with PBST (PBS with 0.1% Triton X-100, Carl Roth) for 10 min. Following three washes in PBS, cells were blocked with 3% BSA (Carl Roth) in PBST for 30 min at room temperature. Primary antibodies were diluted in blocking solution and incubated with cells overnight at 4 °C. After three washes in PBS, cells were incubated with Alexa Fluor-labeled secondary antibodies diluted with 1% BSA in PBS for 1 h at room temperature. Subsequently, cells were washed three times with PBS, and mounted onto microscope slides with VECTASHIELD Antifade Mounting Medium (Bio-Techne) containing 4’,6-diamidino-2-phenylindole (DAPI). Cells were imaged using an LSM800 confocal microscope (Zeiss, Oberkochen, Germany) with a 63× oil immersion objective or an Epi-Scope1-Apotome fluorescence microscope (Zeiss) with a 20× objective. ImageJ and Zeiss ZEN 3.1 (blue edition) software were used for image processing and analysis.

### Flow cytometry

T cell viability and transduction efficiency, surface expression of NKG2D on T cells, T cell subsets, and surface expression of NKG2DLs on B16F10 cells and mouse primary cells (MEFs and mouse AST) were analyzed by FACS. Cells (1 × 10^6^ cells/sample) were harvested, washed twice with ice-cold PBS, and resuspended in 100 μl of ice-cold PBS containing 2% BSA. For T cell viability assay, viability staining was performed prior to antibody staining using a cell viability dye (Viobility 405/452 Fixable Dye, Miltenyi Biotec, #130-109-816) according to the manufacturer’s instructions. Subsequently, cells were stained with labeled antibodies for 20–30 min, according to the manufacturer’s instructions, at 4 °C in the dark. After incubation, cells were washed three times with ice-cold PBS, and accessed on a FACS instrument (Becton Dickinson (BD) Celesta, Franklin Lakes, NJ, USA). Cell sorting was done on a FACS sorter (BD FACSAria III). Data were analyzed using the FlowJo v.10 software (BD).

### Senescence induction

For genotoxic stress-induced senescence, P1–P3 MEFs were treated with 10 µM ETO (Merck) for 48 h, then washed with Dulbecco’s phosphate-buffered saline (DPBS; Thermo Fisher Scientific) and harvested 5 days later. P1–P2 mouse astrocytes were treated with 5 µM ETO for 24 h, then washed and harvested 2 days later. For oxidative stress-induced senescence, P1–P3 MEFs and P1–P2 mouse astrocytes were treated with 200 μM H2O2 (Merck) for 2 h, then washed with DPBS. H2O2 treated MEFs were cultured in complete medium for an additional 7 days, and H2O2 treated mouse astrocytes were cultured in complete medium for an additional 3 days. P1–P3 proliferating cells treated with 0.1% dimethyl sulfoxide (Merck) served as vehicle controls.

### SA-β-gal staining

SA-β-gal activity was measured using the Senescence β-Galactosidase Staining Kit (Cell Signaling Technology, #9860) in accordance with the manufacturer’s protocol. Cells were imaged under bright field using the Epi-Scope1-Apotome microscope (Zeiss) with a 10× objective. ImageJ software was used for image analysis.

### RNA extraction, reverse transcription, and quantitative real-time PCR

Total RNA was extracted from cells using the RNeasy Mini Kit (Qiagen, Hilden, Germany, #74104) according to the manufacturer’s protocol. 1 000 ng of isolated mRNA was reverse-transcribed with the High-Capacity cDNA Reverse Transcription Kit (Thermo Fisher Scientific). Quantitative real-time PCR (qPCR) was carried out using the PowerUP SYBR Green Master Mix (Thermo Fisher Scientific) on a QuantStudio6 Real-Time PCR System (Thermo Fisher Scientific). The cycle threshold (CT) value of each target gene was normalized to the corresponding CT value of β-actin. Primer sequences used are listed in Supplementary Table 2.

### Cytotoxicity assay

To quantify the cytotoxicity of NKG2D-CAR T cells against NKG2DLs highly expressing B16F10 cells and mouse senescent cells, a real-time cytotoxicity assay was carried out. Briefly, target cells were seeded as 3000–5000 cells per well in six replicates in 96-well black plates with a clear bottom (Greiner, Kremsmünster, Austria) in RPMI complete medium. After 24 h, cell culture medium was removed, and 5 μg/ml Calcein-AM (Merck, #56496) in RPMI was added into the plates with target cells and incubated for 30 min at 37 °C in a 5% CO2 incubator. UN-T cells or NKG2D-CAR T cells were resuspended at the indicated dilutions in RPMI complete medium. Then the Calcein-AM-staining medium was removed, and target cells were washed with PBS. 100 μl of effector cell suspension were added into each well in 96-well plates at different E: T ratios. Subsequently, the plate was spun at 200 × *g* for 2 min and incubated in the IncuCyte S3 at 37 °C in 5% CO2 for 4 h (B16F10 cells) or 8 h (mouse primary cells). Live images were acquired every 2 h with a 4× objective for whole-well image to capture the changes in green fluorescence, which indicates the viable Calcein-AM positive target cells. ImageJ software was employed for viable cell counting. To minimize variations in background fluorescence intensity resulting from the gradual release of Calcein-AM during the co-culture period, a consistent background of 20 pixels was subtracted from each image. Subsequently, RGB images were converted to greyscale for cell counting. The binary processing was performed using an adaptive threshold, accompanied by hole-filling procedures to complete cellular structures. To refine the segmentation, a watershed algorithm was applied to separate closely clustered cells. The resulting binary masks were then converted into an image of particles, with all particles sized within the range of 0.003 to Infinity (pixel^2) considered as cells and counted accordingly.

### Statistics analysis and experimental design

Samples were pseudorandomly assigned to experimental or control groups. All experiments were conducted blind to experimental group assignment. All statistical analyses were performed using GraphPad Prism version 9.0 (GraphPad Software, San Diego, CA, USA). Data were presented as mean ± SEM or mean ± SD. Statistical differences between two groups were analyzed using unpaired two-tailed Student’s t-test, and statistical differences among three or more groups were analyzed by one-way or two-way analysis of variance (ANOVA) followed by Tukey’s post-hoc tests. Statistical significance was defined as *P < 0.05; **P < 0.01; ***P < 0.001; ****P < 0.0001; ns = not significant. Figures were prepared using Illustrator v 26.0 (Adobe) and Biorender.com.

### Supplementary information

Supplementary information

Supplementary Figure 1

Full length uncropped western blots

## Data Availability

All data are available from the corresponding author upon request.
