# Supplementary material for: Targeting senescent cells with NKG2D-CAR T cells
Source: Cell Death Discov. 2024 May 4;10:217. doi: 10.1038/s41420-024-01976-7 (PMC11069534; doi:10.1038/s41420-024-01976-7)
Supplement: Supplementary file 1 — Supplementary information [file 41420_2024_1976_MOESM1_ESM.docx]

**Supplementary Materials**

**Targeting senescent cells with NKG2D-CAR T cells**

Yushuang Deng^1^, Avadh Kumar1^,2^, Kan Xie1, [Kristina Schaaf](https://pubmed.ncbi.nlm.nih.gov/?term=Schaaf%20K%5BAuthor%5D)1, Enzo Scifo1, Sarah Morsy1^,3^, Tao Li^4^, Armin Ehninger^3^, [Daniele Bano](https://pubmed.ncbi.nlm.nih.gov/?term=Bano%20D%5BAuthor%5D)^5^, Dan Ehninger1*

^1^Translational Biogerontology Lab, German Center for Neurodegenerative Diseases (DZNE), Venusberg-Campus 1/99, 53127 Bonn, Germany.

^2^Lonza Netherlands B.V., Geleen, [Urmonderbaan 20-B, 6167 RD Geleen, Netherlands](https://www.google.com/url?sa=t&rct=j&q=&esrc=s&source=web&cd=&cad=rja&uact=8&ved=2ahUKEwi6n9GI5oeEAxW19gIHHRjMBygQ4kB6BAgkEAM&url=%2Fmaps%2Fplace%2F%2Fdata%3D!4m2!3m1!1s0x47c0c6f23974c493%3A0xb0cdf70cd75d9847%3Fsa%3DX%26ved%3D2ahUKEwi6n9GI5oeEAxW19gIHHRjMBygQ4kB6BAgSEAA&usg=AOvVaw0SF2hQ8Fe_AwzP5m1BLPZ1&opi=89978449).

^3^AvenCell Europe GmbH, Tatzberg 47, 01307 Dresden, Germany.

^4^Department of Neurodegenerative Disease and Geriatric Psychiatry/Neurology, University of Bonn Medical Center, 53127 Bonn, Germany.

^5^Aging and Neurodegeneration Lab, German Center for Neurodegenerative Diseases (DZNE), Venusberg-Campus 1/99, 53127 Bonn, Germany.

*Correspondence to: [Dan.Ehninger@dzne.de](mailto:Dan.Ehninger@dzne.de)

**This file includes:**

Supplementary Figure legends

Supplementary Tables 1-2

**Supplementary Figure legends**

**Supplementary Figure 1. Validation of isolated mouse astrocytes by GFAP staining.** Isolated mouse forebrain astrocytes at early passages (P1-P2) were immunostained using an anti-GFAP (green) antibody and DAPI (red). Scale bar = 50 μm. GFAP, glial fibrillary acidic protein; DAPI, 4’,6-diamidino-2-phenylindole.

**Supplementary Table 1. List of PCR primer sequences used for vector construction**

Restriction sites were underlined.

| **Primer name** | **Forward** | **Reverse** |
| --- | --- | --- |
| mNKz-CAR | ATAGTCGACACCATGAGAGCAAAATTCAGCAGGAGTGCAG | ATAGCGGCCGCTCGAGTCCGGACACCGCCCTTTTCATGCAGATGTACGTGTTTAG |
| mNKz-CAR-FLAG | ATTTGCGGCCGCATGAGAGCAAAATTCAGCAGGAGTG | CTAGCTAGCTTACTTGTCATCATCGTCCTTGTAGTCCACCGCCCTTTTCATGCAGATGTAC |
| PiggyBac_H60a | TGATAGCGGCCGCATGGCAAAGGGAGCCACCAGCAAGAG | TAAGACGCGTTAGCTGGTAATGAGGACTGCAGAAGAAG |
| PiggyBac_Rae1β | TAATAGCGGCCGCATGGCCAAGGCAGCAGTGACCAAG | CACGACGCGTCATCGCAAATGCAAATGCAAATAATAAAG |
| PiggyBac_Mult1 | TGATAGCGGCCGCATGGAGCTGACTGCCAGTAACAAGGTC | TACGACGCGTTGGGATCCCATCAATATCGTCTGAAGTCAACAGC |
| PiggyBac_EF1α | TGTAAGAATTCAGTTTGGACTAGTCGTGAGGCTCCGG | GTAACTAAGCTTACCGGTTCTAGAGCGGCCGCTTCACGACACCTGAAATGGAAG |
| PiggyBac_T2A-Neo-SV40 PolyA | AATATCTAGAGACGTCACGCGTGAGGGCAGAGGAAGTCTTCTAACATGCGGTGACGTGGAGGAGAATCCCGGCCCTGCTAGCATGATTGAACAAGATGGATTGCACGCAGGTTCTCC | CAGCAAGCTTTAAGATACATTGATGAGTTTGGACAAAC |

mNK, murine Natural killer group 2D; CAR, chimeric antigen receptor; H60a, histocompatibility antigen 60a; Rae1β, retinoic acid early transcript 1β; Mult1, murine UL16-binding-protein-like transcript 1; EF1α, elongation factor 1-alpha; T2A, thosea asigna virus 2A; Neo, neomycin resistance; SV40 PolyA, simian virus 40 PolyA.

**Supplementary Table 2. List of primer sequences used for real-time quantitative PCR analyses**

| **Gene name** | **Forward** | **Reverse** |
| --- | --- | --- |
| H60a (Mus musculus) | AACCATTGCCTGATTCTGAGC | TGGGACAAATCAGCACACATC |
| H60b (Mus musculus) | TCAGAAGGGATGAGGAACCAG | GTTGATGGCCCAGAATCCAC |
| H60c (Mus musculus) | GCTGCCTCAACAAATCGTCG | ATCAACCCATCAAAGGGGCT |
| Rae1α (Mus musculus) | GGGAGACAGCAAATGCCACT | AGGAATTTGGCCCTGGCTTT |
| Rae1β (Mus musculus) | GCAAATGCCACTGAAGTGAAG | CCATTGGTCTTGTGAGTGTCC |
| Rae1δ (Mus musculus) | TCCTACCCCAGCAGATGAAGT | TTCAGTGGCATTTGCTGTCTC |
| Rae1ε (Mus musculus) | TCCTACCTCAGCAGACCTTCC | TCCTGGCACAAATCGTTCAGA |
| Mult1 (Mus musculus) | TTGACAGTGCCTGAGACGTG | TCGTCTGAAGTCAACAGCACA |
| Actb (Mus musculus) | CCCTGAAGTACCCCATTGAAC | CCATGTCGTCCCAGTTGGTAA |

H60, histocompatibility antigen 60; Rae1, retinoic acid early transcript 1; Mult1, murine UL16-binding-protein-like transcript 1; Actb, actin beta.
