# Supplementary figures and images for: Targeting senescent cells with NKG2D-CAR T cells
Source: Cell Death Discov. 2024 May 4;10:217. doi: 10.1038/s41420-024-01976-7 (PMC11069534; doi:10.1038/s41420-024-01976-7)

Supplementary Figure 1

GFAP

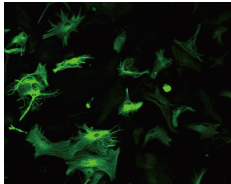

DAPI

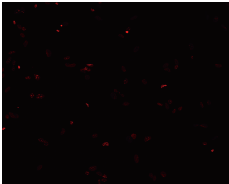

Merge

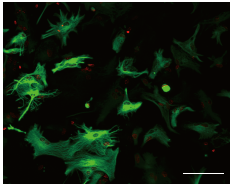

Supplement: Supplementary file 2 — Supplementary Figure 1 [file 41420_2024_1976_MOESM2_ESM.pdf]

**Fig. 1B**

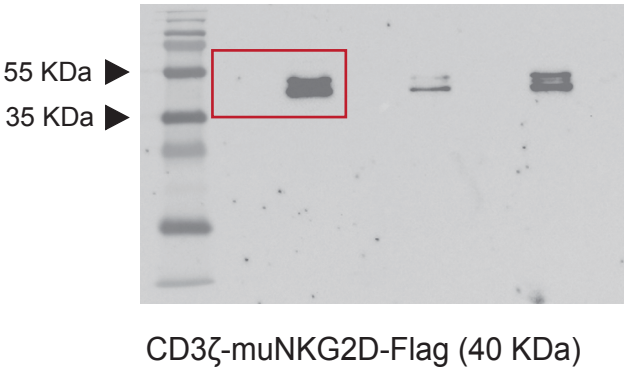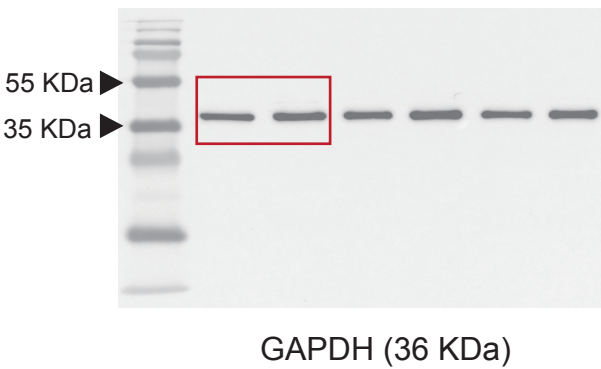

**Fig. 3B (MEF)**

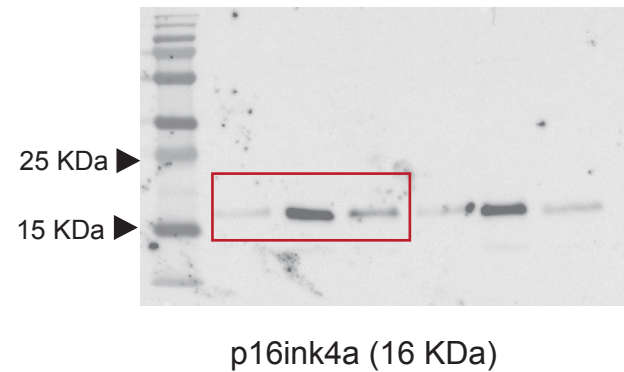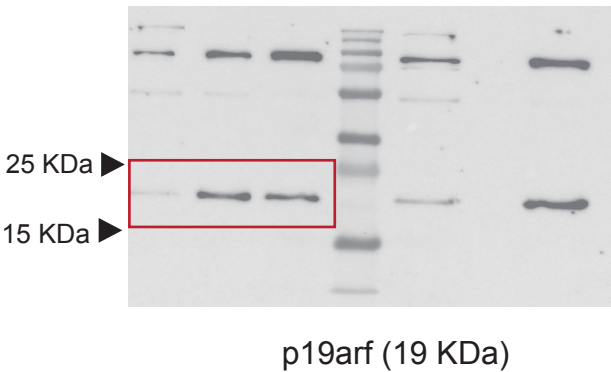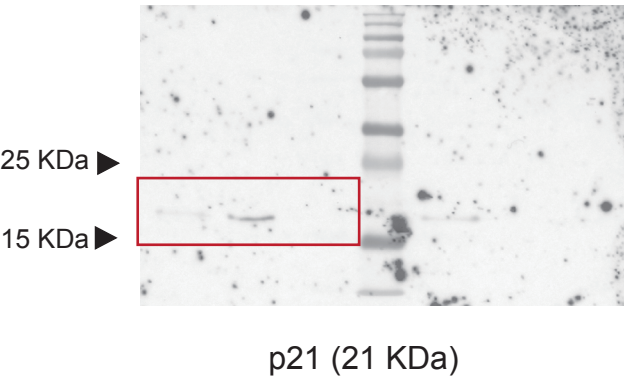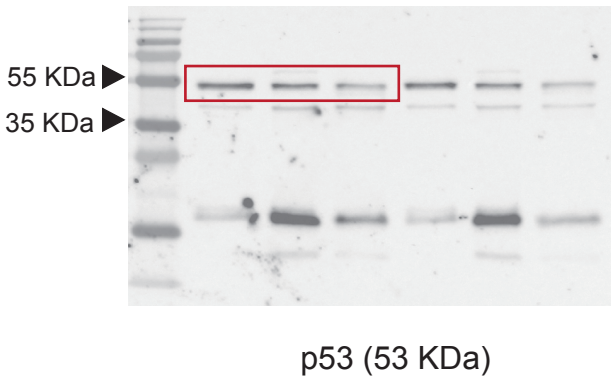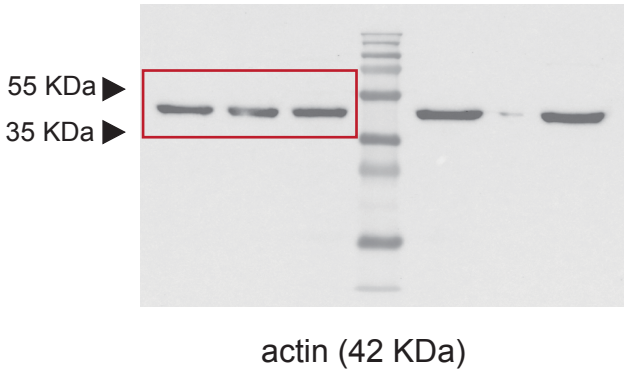

**Fig. 3B (AST)**

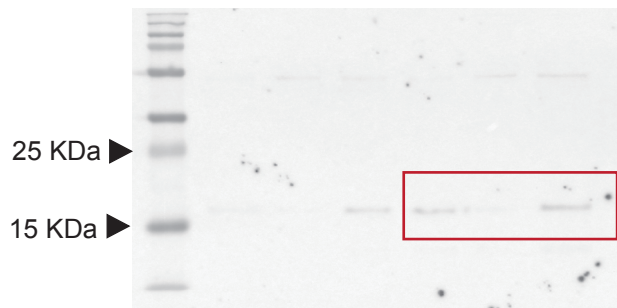

p16ink4a (16 KDa)

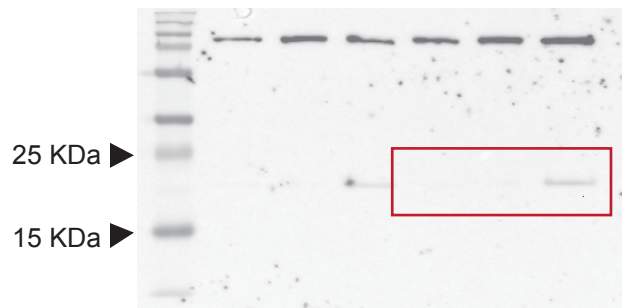

p19arf (19 KDa)

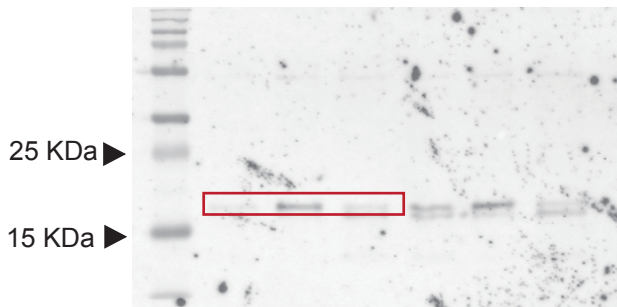

p21 (21 KDa)

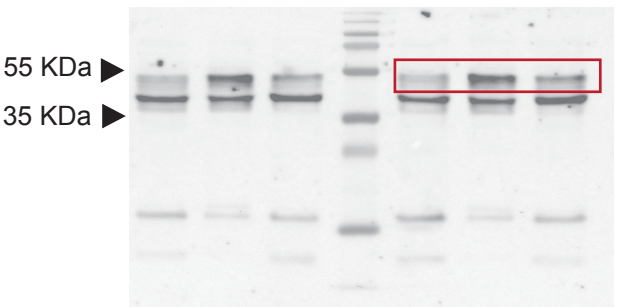

p53 (53 KDa)

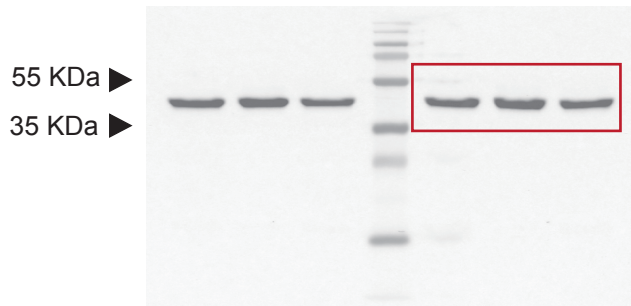

actin (42 KDa)

Supplement: Supplementary file 3 — Full length uncropped western blots [file 41420_2024_1976_MOESM3_ESM.pdf]
